# New Insight into Biofilm Formation Ability, the Presence of Virulence Genes and Probiotic Potential of *Enterococcus* sp. Dairy Isolates

**DOI:** 10.3389/fmicb.2018.00078

**Published:** 2018-01-30

**Authors:** Nikola Popović, Miroslav Dinić, Maja Tolinački, Sanja Mihajlović, Amarela Terzić-Vidojević, Svetlana Bojić, Jelena Djokić, Nataša Golić, Katarina Veljović

**Affiliations:** ^1^Laboratory for Molecular Microbiology, Institute of Molecular Genetics and Genetic Engineering, University of Belgrade, Belgrade, Serbia; ^2^HITTest Doo, Belgrade, Serbia

**Keywords:** *Enterococcus* sp., dairy isolates, virulence, biofilm formation, antibiotic resistance, adhesion, pathogen exclusion

## Abstract

Enterococci have controversial status due to their emerging role in nosocomial infections and transmission of antibiotic resistance genes, while some enterococci strains are used as probiotics for humans and animals and starter cultures in dairy industry. In order to improve our understanding of factors involved in the safe use of enterococci as potential probiotics, the antibiotic susceptibility, virulence and probiotic traits of 75 dairy enterococci isolates belonging to *Enterococcus durans* (50), *En. faecium* (15), *En. faecalis* (6), *En. italicus* (3), and *En. hirae* (1) were evaluated. The results revealed that ciprofloxacin resistance and biofilm formation are correlated with isolates originated from Golija mountain (Serbia), while gelatinase activity was more common in isolates from Prigorje region (Croatia), pointing to uncontrolled use of antibiotics and anthropogenic impact on dairy products' microbiota in these regions. The virulence genes were sporadically present in 13 selected dairy enterococci isolates. Interestingly, biofilm formation was correlated with higher ability of strains to reduce the adhesion of *E. coli* and *Salmonella* Enteritidis to HT29-MTX cells. To our knowledge this is the first study reporting the presence of the *esp* gene (previously correlated with pathogenesis) in dairy enterococci isolates, mostly associated with the genes involved in adhesion property. Hence, the results of this study revealed that the virulence genes are sporadically present in dairy isolates and more correlated to adhesion properties and biofilm formation, implicating their role in gut colonization rather than to the virulence traits.

## Introduction

Enterococci are the most controversial group of lactic acid bacteria (LAB). *Enterococcus faecalis* and *En. faecium* are found to be the most frequent enterococci species in human infections and represent the third most frequently isolated multidrug resistant nosocomial pathogens from bacteraemia (Soares et al., 2014; Van Tyne and Gilmore, 2017). The virulence factors found in enterococci include gelatinase (GelE) activity, the presence of enterococcal surface protein (Esp), aggregation factor (Agg), hyaluronidase (Hyl), and cytolysin (Cyl, β-hemolysin) (Anderson et al., 2015). The virulence factors contribute to fitness and persistence of enterococci in nosocomial infections. Enterococci have an extraordinary potential to form biofilm highly resistant to antibiotics, especially in specific environmental conditions, such as urinary tract and oral cavity (Soares et al., 2014). However, the biofilm formation was shown to be more related to the adhesion properties, a desirable probiotic characteristic, than to the virulence genes (Pieniz et al., 2015).

On the other hand, enterococci are commensals in gastrointestinal tract (GIT) as well as important constituents of traditional Mediterranean food of animal origin and contribute to their characteristic taste and flavor (Banwo et al., 2013). Despite of the controversial status, recently the probiotic potential of enterococci has been acknowledged (Franz et al., 2011; Kreuzer et al., 2012; Banwo et al., 2013; Pieniz et al., 2015). Interest in the health-promoting potential of enterococci has significantly increased due to their robustness and ability to survive harsh conditions such are present in the GIT (FAO-WHO, 2016). Other important criteria for probiotics are adhesion to intestinal epithelial cells (IEC) important for colonization of intestinal mucosa, persistence in the intestine and the competitive exclusion of pathogens (FAO-WHO, 2016; Živković et al., 2016).

Our previous results based on analysis of 50 antibiotic resistant enterococci in environmental waters of Belgrade (Serbia) suggested the anthropogenic impact on the spread of antibiotic resistance genes among enterococci in urban surface waters that is most likely attributed to the uncontrolled use of antibiotics (Veljović et al., 2015). However, our work related to dairy enterococci isolates revealed that autochthonous dairy products from the Western Balkan Countries (WBC) region are a rich source of novel enterococci strains with substantial genetic, metabolic and technological potential, although the presence of antibiotic resistance and virulence genes were revealed, pointing out that the determination of the safety status of each potential probiotic enterococci strain is mandatory in order to prevent the potential risks of their administration to humans and animals (Terzić-Vidojević et al., 2015).

## Materials and methods

### Bacterial strains, media, and growth conditions

The 75 previously isolated enterococci dairy isolates belonging to five distinct species were used (Table S1; Terzić-Vidojević et al., 2015). Enterococci were grown in M17 broth (Merck, GmbH, Darmstadt, Germany) supplemented with glucose (0.5% w/v) (GM17) at 37°C. *Escherichia coli* ATCC 25922, *Salmonella* Enteritidis 654/7E and other indicator strains (Table S2) were cultivated in Luria-Bertani broth (LB), containing 0.5% NaCl, 0.5% yeast extract (Torlak, Belgrade, Serbia), and 1% tryptone (Torlak) at 37°C. Corresponding agar plates were prepared by adding agar (1.7% w/v, Torlak) into each broth.

### Haemolytic and gelatinase activity assays

Haemolytic activity was determined on Columbia Blood Agar (Oxoid Limited, Hampshire, UK) containing 5% (v/v) defibrinated horse blood after 48 h of incubation at 37°C (Valenzuela et al., 2009). The phenotypic assay of gelatinase activity was determined on plates agar containing 3% (w/v) gelatin (Difco, Becton Dickinson, New Jersey, United States) (Lopes et al., 2006).

### Biofilm formation

Biofilm formation assay was performed as described previously (Macovei et al., 2009) with minor modifications. Biofilm production was quantified using 0.1% crystal violet (HIMedia, Mumbai, India) and measuring absorbance at 595 nm using Plate Reader Infinite 200 pro (MTX Lab Systems, Vienna, Austria).

### Susceptibility testing

Minimal inhibitory concentrations (MICs) were determined by microdilution testing following (CLSI, 2015) criteria. Susceptibility was tested against: ampicillin (8–16 μg/ml), vancomycin (4–32 μg/ml), erythromycin (0.5–8 μg/ml), tetracycline (4–16 μg/ml), ciprofloxacin (1–4 μg/ml), nitrofurantoin (32–128 μg/ml), chloramphenicol (8–32 μg/ml), linezolid (2–8 μg/ml), and gentamicin (>500 μg/ml). Additionally, according to European Food Safety Authority (EFSA, Panel, 2012) guidance for *Enterococcus faecium*, susceptibility of *En. faecium* strains was tested against: ampicillin (1–2 μg/ml), gentamicin (16–32 μg/ml), kanamycin (512–1024 μg/ml), streptomycin (64–128 μg/ml), clindamycin (2–4 μg/ml) and tylosine (2–4 μg/ml). Microdilution tests were performed in GM17 (Merck, GmbH). Cell density was monitored after 24 h incubation at 37°C at 595 nm using Plate Reader Infinite 200 pro (MTX Lab Systems).

### PCR detection of virulence determinants

The total DNA of 13 antibiotic-susceptible and without haemolytic and gelatinase activity enterococci strains was used in PCR reactions to detect the presence or absence of genes for virulence determinants. The primer sequences of the target genes, the expected amplicon sizes and annealing temperatures are given in Table S3.

### Antimicrobial activity assay

Production of antimicrobial compounds produced by enterococci was tested by the deferred antagonism method using various indicator strains (Lozo et al., 2004). Indicator strains used in this assay are listed in Table S2. The presence of bacteriocin-encoding genes was studied by PCR amplification, with primers for well-known enterococcal bacteriocins using total genomic DNA from strains (Table S3).

### MTT assay

The cytotoxicity of enterococci on HT29-MTX cell line was evaluated through a microculture tetrazolium [MTT, 3-(4, 5-dimethylthiazol-2-yl)-2,5-diphenyltetrazolium bromide] assay (Mosmann, 1983). The colonocyte-like cell line HT29-MTX, used in MTT assay, was kindly supplied by Dr. T. Lesuffleur (INSERM UMR S938, Paris, France; Lesuffleur et al., 1990). After 24 h of post seeding (40–60% confluency), different concentrations (ratio 1:1 and 10:1 bacteria: eukaryotic cell) of the filtered supernatants (using 0.22 μm Nalgene syringe filter units, Sarstedt, Germany) and UV-irradiated non-viable bacterial cells were added to the eukaryotic cells. All treated cells were then incubated for 24 and 48 h. After the treatment, MTT was added at the final concentration of 0.5 mg/ml. The plates were incubated for 4 h in 5% CO_2_ at 37°C in dark conditions. Formazan crystals created in MTT-exposed live cells were dissolved by adding 10% sodium–dodecyl sulfate (SDS) in 0.01% HCl. The wells were then incubated at 37°C overnight. Adsorption of the dissolved formazan crystals was measured using Plate Reader Infinite 200 pro at 570 nm. The results were expressed as the percentage of optical density (metabolic activity) compared to the control (cultures of non-treated cells), used as 100% as follows:

Metabolic activity (%) = (O.D. of treated cells/(O.D. of non-treated cells) × 100.

### Survival in simulated gastrointestinal tract

Survival in chemically simulated gastrointestinal tract was performed using an *in vitro* test as described previously (Sánchez et al., 2010). The percentage of survival was calculated from the viable counts recovered after each chemically simulated GIT step with respect to the initial counts (%) = (CFU/mL recovered bacteria/CFU/mL initial bacteria) × 100.

### Mucin adhesion assay

Mucin adhesion assays were performed in 96-well polystyrene microtitre plates (Tissue Culture Plate, Sarsted, Newton, USA) according to the method of (Valeriano et al., 2014) with minor modifications: The wells of microtitre plates were coated at 4°C for 24 h with 200 μl of porcine stomach mucin type II (Sigma, Germany). Mucin was resuspended (1 mg/ml) in phosphate-buffered saline (PBS) buffer (pH 7.0) and the same volume of PBS was added to control wells. Wells were washed twice with 200 μl PBS and incubated with 100 μl (20 mg/ml) bovine serum albumin (BSA) (Sigma) for 2 h at 4°C. Wells were again washed twice with 200 μl of PBS to remove unbound BSA. Approximately 100 μl of bacterial suspension (10^8^ CFU/ml) was washed and suspended in PBS (pH 7.0) and added to the wells. Plates were incubated at 37°C for 2 h. After incubation, wells were washed three times with 200 μl PBS to remove unbound bacteria. Another 200 μl of 0.5% (v/v) Triton X-100 (Sigma) was then added to isolate attached bacteria. The viable cell count expressed as CFU/ml was determined in all cases by plating on GM17. Percentage adhesion was calculated from the viable counts adherent to the mucin with respect to the initial counts (%) = (CFU/mL recovered bacteria/CFU/mL initial bacteria) × 100.

### Adhesion to HT29-MTX cell line

For adhesion experiments, 13 ± 1 day-old cellular monolayers of HT29-MTX were used according to Živković et al. (2016). Cellular monolayers were also carefully washed and bacterial suspensions were added at a ratio of about 10:1 (bacteria: eukaryotic cell). Adhesion experiments were carried out for 1 h at 37°C, 5% CO_2_. The adhesion was calculated as (%) = (CFU/mL adhered bacteria/CFU/mL added bacteria) × 100.

### Pathogen exclusion assay

The capability of reference strains *E. coli* ATCC25922 and *Salmonella* Enteritidis 654/7E to adhere to the intestinal epithelium in the presence and absence of enterococci was tested according to Živković et al. (2016). The bacterial suspensions containing *E. coli* or a combination of *E. coli* and enterococci (ratio 1:1) and *Salmonella* Enteritidis or a combination of *Salmonella* Enteritidis and enterococci (ratio 1:1) were independently added to the HT29-MTX monolayers at a ratio of 10:1, (bacteria: eukaryotic cell) and incubated at 37°C, with 5% CO_2_ for 1 h. The percentage of adhesion was calculated as follows: 100 × CFU/mL bacteria adhered to HT29-MTX monolayers / total CFU/mL bacteria added (adjusted for dilution). Numbers of bacteria were determined via viable cell count expressed as CFU/ml determined by plating on GM17. To determine the capability of the enterococci to inhibit the adhesion of *E. coli* and *Salmonella* Enteritidis to HT29-MTX monolayers, data were referred to that obtained with the *E. coli* and *Salmonella* Enteritidis alone (i.e., 100% adhesion).

### Statistical analysis

All experiments were designed based on a completely random design and statistical differences in multiple groups were determined by one-way ANOVA followed by multiple mean comparisons Duncan's test. A *p* ≤ 0.05 was here considered statistically significant. Data were presented as the mean ± standard deviation of three independent replicates. Rules of association between geographic origin of strains and different phenotypic characteristics were mined as described in Hahsler et al. (2005), using R package “arules.” Statistical significance of individual associations were evaluated through “HyperConfidence” measure, and corrected for multiple comparisons according to Benjamini-Hochberg method. DNA 16S sequences of 75 selected strains were aligned with Clustal Omega web tool (McWilliam et al., 2013), pairwise Kimura's 2-parameters distance were calculated as implemented in the R package “ape” (Paradis et al., 2004), and clustered using method “complete linkage.” Hierarchical clustering of bacterial phenotypes was based on Jaccard distance, same agglomeration method, and the similarity of two trees was evaluated through Baker's gamma index, using R package “dendextend” (Galili, 2015).

## Results and discussion

Due to the controversial status of enterococci, the focus of this study was the evaluation of presence of antibiotic resistance, virulence genes, and biofilm formation in 75 previously isolated dairy enterococci from WBC region (Table S1, Figure S1; Terzić-Vidojević et al., 2015) and their correlation with the origin and phylogenetic relatedness. To our knowledge, this is the first study combining data on virulence and probiotic traits in order to improve our understanding of the dairy products as a potential reservoir of virulent strains, as well as the implications for the safety use of dairy enterococci isolates as potential probiotics.

### Virulence traits, antibiotic susceptibility and cytotoxic effect

The important features of enterococcal virulence are hemolytic and gelatinase activities (Banwo et al., 2013). The results revealed that 14 out of 75 strains (18.7%) exhibit haemolytic activity: 33.3% *En. faecalis* (2 out of 6) and *En. italicus* (1 out of 3), 20% *En. durans* (10 out of 50), and 6.7% *En. faecium* (1 out of 15). *En. hirae* strain did not exhibit haemolytic activity on blood agar plates after 48 h incubation (Table S1). The activity of gelatinase, an extracellular zinc metallo-endopeptidase, able to hydrolyse gelatin, casein and hemoglobin (Koch et al., 2004; Banwo et al., 2013), occurred in 5 out of 50 (10%) *En. durans* isolates (Table S1).

Although all 75 strains were previously determined as antibiotic susceptible, according to the results obtained by disc diffusion method (Terzić-Vidojević et al., 2015), the antibiotic susceptibility to nine of the most clinically relevant antibiotics of 56 enterococci strains without haemolytic and gelatinase activity were additionally tested by microdilution test, according to CLSI. In total, only 23 out of 56 strains were antibiotic susceptible, while 57% were resistant to ciprofloxacin (27 strains) or gentamicin (6 strains). According to EFSA, all previously susceptible *En. faecium* were resistant to low concentration of ampicillin, gentamicin and streptomycin. In general, *Enterococcus* strains were found to be moderately sensitive to streptomycin, gentamicin and ciprofloxacin and are generally regarded as intrinsically resistant to low levels of gentamicin, although a high-level gentamicin resistance was detected in many dairy isolates (Giraffa, 2003; Hummel et al., 2007; Banwo et al., 2013). Hence, the observed high levels of ciprofloxacin and gentamicin resistance among our dairy enterococci isolates are in accordance to literature data and possibly reflect the extensive use of these antibiotics in animal husbandry in WBC region.

The 13 antibiotic susceptible strains were further tested for presence of the virulence genes encoding aggregation factor (*agg*), collagen adhesin (*ace*), cytolysin (c*ylA*), enterococcal surface protein (*esp*), cell wall adhesins (*efaA*^*fs*^, and *efaA*^*fm*^), gelatinase (*gelE*), hyaluronidase (*hylN*), serine protease (*sprE*), response regulator (*fsrA*), signaling peptide (*fsrB*), and histidine kinase (*fsrC*) (Table 1). Ten strains (76.9%) were positive for the *efaA*^*fs*^ gene and nine strains (69.2%) were positive for the *agg* gene. Seven strains (53.8%) were positive for the *sprE* gene, while six strains (46.2%) were positive for *esp* and *efaA*^*fm*^ genes. Three strains (23.1%) were positive for the *fsrA* gene, while two strains (15.4%) were positive for the *gelE* gene. One strain (7.7%) was positive for *fsrC* gene, while *fsrB* gene was not detected.

**Table 1:** Presence of the virulence genes, genes for biofilm formation and bacteriocin production.

**Species/Strain**	**Adhesins**				**Biofilm formation**					**Bacteriocins**		
	***agg***	***esp***	***efaA^*fs*^***	***efaA^*fm*^***	***fsrA***	***fsrB***	***fsrC***	***gelE***	***spreE***	***entA***	***ent1071***	***bac31***
*En. durans* BGGO8-25												
*En. durans* BGGO8-26												
*En. durans* BGGO8-30												
*En. durans* BGGO9-30												
*En. durans* BGRE2-40												
*En. hirae* BGRE2-48												
*En. durans* BGVL2a-53												
*En. durans* BGVL2a-55												
*En. italicus* BGTRK4-42												
*En. durans* BGTRS10-45												
*En. durans* BGBU1-46												
*En. durans* BGPT2-84												
*En. durans* BGAL3-19												

*The shaded areas reflect the presence of the respective genes*.

It is noteworthy that the c*ylA* gene encoding CylA serine protease involved in processing and activation of cytolysin (also called haemolysin), bacterial toxin with β-haemolytic properties in humans, as well as the *hylN* gene, encoding hyaluronidase, degradative enzyme associated with tissue damage (Anderson et al., 2015), were not detected. Interestingly, the gelatinase activity was not detected in any of the strains positive for the presence of the *gelE* gene. The results are in concordance to the literature data suggesting that *gelE* genes could be silent (Franz et al., 2001). The presence of the *gelE* gene was sporadically correlated with the genes for adhesion properties and serine protease. Both gelatinase and serine protease are involved in enterococcal pathogenesis providing nutrients to the bacteria by degradation of host tissue, but also have the role in biofilm formation (Fisher and Phillips, 2009). The *sprE* gene, encoding serine protease, is located directly downstream and co-transcribed with the *gelE* gene and they are together regulated by quorum-sensing system encoded by the *fsr* (fecal streptococci regulator) locus (Fisher and Phillips, 2009). It was shown previously that virulence genes were more prevalent in clinical than in food isolates (Medeiros et al., 2014). Although, the high percentage of virulence genes in our dairy isolates is in agreement with the results obtained on isolates from raw milk and cheese where high abundances of *asa1, gelE*, and *efaA* genes together with high percentage of the strains with haemolytic activity was scored (Moraes et al., 2012).

The intestinal epithelium is delicately responsive to modulations by commensal and pathogenic bacteria and is dependent on the integrity of intestinal epithelial barrier (Howarth and Wang, 2013). In order to determine the possible cytotoxic effects, the HT29-MTX cells were exposed to the supernatants of overnight bacterial cultures (soluble bacterial products), as well as UV-irradiated non-viable bacterial cells (surface bacterial cell molecules) of 13 selected isolates. The results of MTT test showed that none of the tested bacteria, their soluble products and surface molecules significantly affect metabolic activity of HT29-MTX cells in applied conditions. The obtained results suggested that none of the tested dairy isolates exhibit cytotoxic effect on intestinal epithelial barrier indicating possible safe status of the strains (Figure S2).

### The gut colonization, adhesion properties and biofilm formation

The gut colonization is the first step in interaction of indigenous bacterial strain with the host. However, in order to be effective in the gut environment the strains should be resistant to conditions present in GIT (Nishiyama et al., 2016). The ability of the 13 antibiotic susceptible strains without haemolytic and gelatinase activity to survive in simulated GIT conditions was tested (Figure S3). Particularly, most of the strains survived well under simulated gastric conditions (94.4–99.8%; Δlog CFU/ml from 0.02 to 0.52) indicating the resistance of the isolates to acidic conditions. The survival rate was either maintained or slightly decreased after the prolonged exposure times to lower bile concentrations (0.3%) (86.1–98.3%; Δlog from −0.02 to 1.03) and pancreatic enzymes (82.7–93.8%; Δlog from −0.51 to 0.89). In general, the mean survival ability of the strains to simulated GIT conditions was observed to be quite high comparable to the previously tested *Lactococcus* sp. and *Lactobacillus* sp. dairy isolates from Western Balkan region (Uroic et al., 2014) pointing to the fact that enterococci species belong to the commensal bacteria in the gut being their regular habitat.

**Figure 1:**
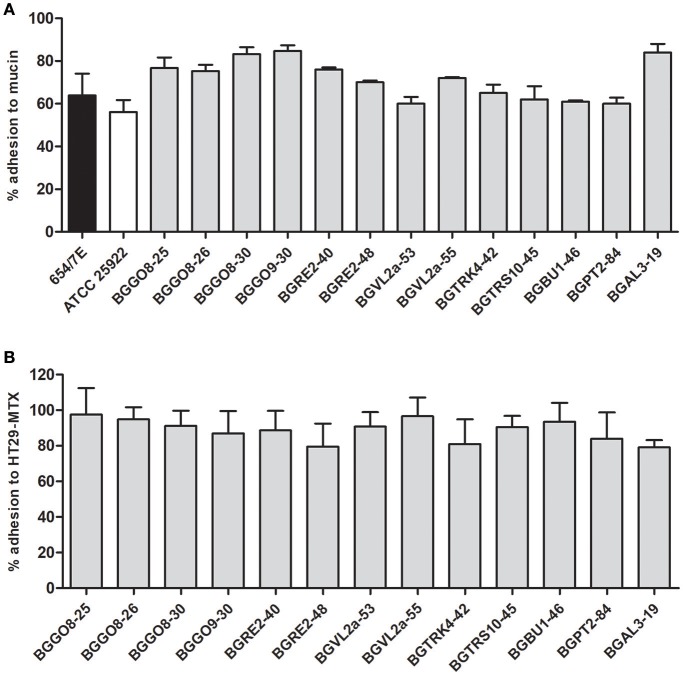
Adhesion of the enterococci strains to mucin **(A)** and the human intestinal epithelial cell line HT29-MTX **(B)**.

Although adhesion properties of pathogenic bacteria are more intensively studied, various cell-surface proteins in probiotic lactic acid bacteria (LAB) and bifidobacteria are shown to have adhesion properties and they have been associated with probiotic activity (Nishiyama et al., 2016). Interestingly, data related to adhesion of enterococci are mostly based on clinical strains and related to virulence determinants (Elhadidy and Zahran, 2014). Our results revealed the high ability of 13 enterococci dairy isolates to bind to mucin (with the average value of 71.88% ± 1.61) and to HT29-MTX epithelial cells (average values of the adhesion to HT29-MTX 88.79% ± 2.32) (Figures 1A,B). Differences in the adhesion were observed among strains. In addition, all 13 isolates exhibit higher relative adhesion to mucin (from 60.6 to 84%) in comparison to *E. coli* ATCC 25922 (56.1%) and *Salmonella* Enteritidis 654/7E (63.9%) (Figure 1A). Interestingly, the majority of tested enterococcal isolates were positive for the *efaA*^*fs*^ gene (76.9%), the *agg* gene (69.2%), the *esp* gene (46.2%), and the *efaA*^*fm*^ gene (46.2%), and associated with the adhesion ability. Aggregation substance, encoded by the *agg* gene, is a pheromone-inducible surface glycoprotein involved in bacterial conjugation and facilitating plasmid transfer, as well as adhesion to an array of eukaryotic surfaces (Koch et al., 2004). The *esp* gene encodes extracellular surface protein, a cell-wall-associated protein that promotes adhesion, colonization and evasion of the immune system, and contributes to biofilm formation (Koch et al., 2004). The virulence genes were determined in *En. faecalis* and *En. faecium* strains of food origin, although the *esp* gene was only observed in the isolates from water, vegetables and raw milk (Anderson et al., 2015). There were no data about the presence of the *esp* gene in isolates of dairy origin (Medeiros et al., 2014). However, in our study the *esp* gene was detected in 6 out of 13 strains (6 *En. durans*). Interestingly, the *esp* gene in these strains was mostly correlated with the *agg, efaA*^*fs*^ and *efaA*^*fm*^ genes involved in adhesion property of the strains and the *sprE* gene. Moreover, in *En. durans* strains BGPT2-84 and BGAL3-19 the presence of the *esp* gene was correlated with biofilm formation ability. The *ace* gene encoding collagen adhesin was not detected in any of the tested isolates.

Besides, biofilm formation has considerable role in enterococcal infections (Mohamed and Huang, 2007). In this study, 30 out of 75 strains (40%) were able to form biofilm (Table S1). Particularly, the biofilm formation was observed in two out of three *En. italicus* (66.7%), three out of six *En. faecalis* (50%), *six* out of 15 *En. faecium* (40%), and 19 out of 50 *En. durans* (38%) strains, respectively, while *En. hirae* strain was not able to form biofilm (Table S1). Although some literature data indicated that biofilm formation capacity was higher in clinical and oral isolates than in the food isolates (Anderson et al., 2015), other authors reported that commensal isolates from human feces exhibit higher biofilm formation ability than endocarditis isolates suggesting that biofilm formation is not exclusively related to the pathogenesis and that it is more associated with adhesion properties, an important feature in gut colonization (Johansson and Rasmussen, 2013; Elhadidy and Zahran, 2014).

### Antimicrobial activity

Finally, the capability to neutralize negative effects of pathogens is one of the crucial beneficial health claims of probiotics. Our results revealed that four *En. durans* strains showed inhibitory effect on *En. faecalis* BG221. No activity was observed against *Salmonella* Enteritidis 654/7E and *E. coli* ATCC 25922. The PCR screening of bacteriocin genes revealed the presence of three bacteriocin genes *entA, ent1071*, and *bac31* in the genomes of studied enterococci strains (Table 1). The results of this study are in agreement with our previous results indicating that natural dairy enterococci isolates from the WBC region are capable of producing bacteriocins (enterocins) with broad spectrum activity (Veljovic et al., 2009; Terzić-Vidojević et al., 2015).

**Figure 2:**
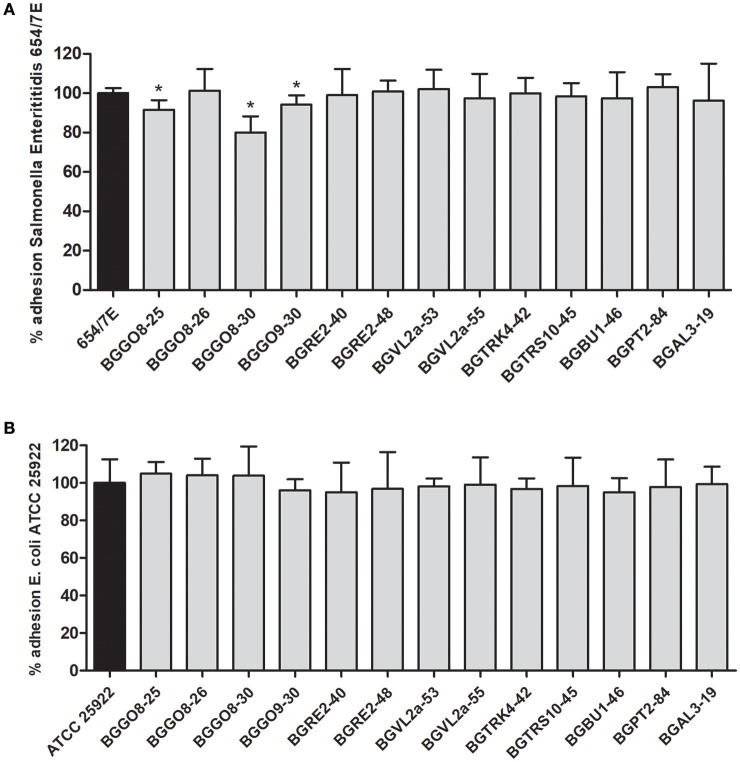
Association of *Salmonella* Enteritidis 654/7E to HT29-MTX cells in the presence of enterococci **(A)** and association of *E. coli* ATCC 25922 to HT29-MTX cells in the presence of enterococci **(B)**. The statistical differences with respect to the control strains are annotated with asterisks (*p < 0.05).

On the other hand, while numerous studies reported the pathogen exclusion by lactobacilli probiotic strains (Živković et al., 2016), the previous results (Rinkinen et al., 2003) indicated that *En. faecium* M74 and *En. faecium* SF273 strains favor the colonization of *Campylobacter jejuni* in dog's intestinum, pointing to the additional risk factor for use of enterococci as probiotics. The results obtained in this study revealed that adhesion of *E. coli* ATCC 25922 and *Salmonella* Enteritidis 654/7E to HT29-MTX was reduced in the presence of 10 tested enterococci strains (Figures 2A,B). The lowest adhesion rate (80.1%) of *Salmonella* Enteritidis 654/7E to the HT29-MTX cells was scored in co-incubation with *En. durans* BGGO8-30 strain. In addition, BGGO9-30 and BGGO8-25 slightly reduced the adhesions of *Salmonella* Enteritidis 654/7E to the HT29-MTX cells (94.1 and 91.5%, respectively).

### Relationship between isolates' origins, phylogenetic relatedness and virulence traits

**Figure 3:**
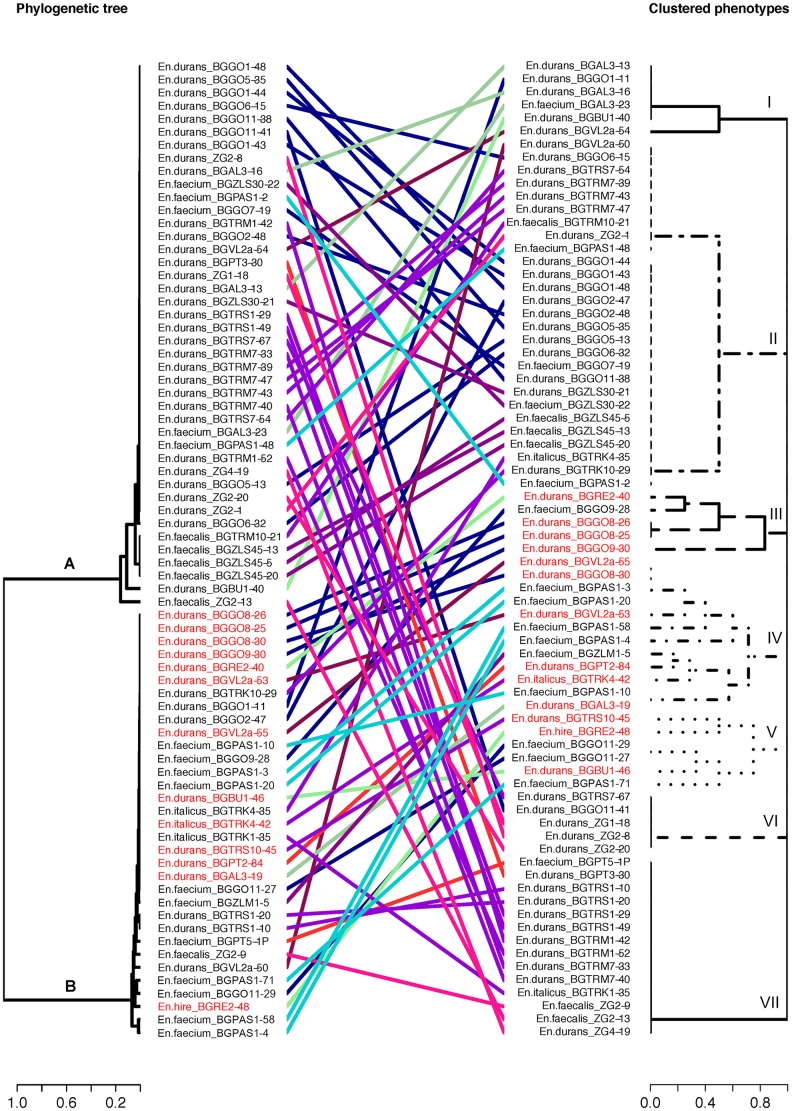
Concordance between enterococci 16S rDNA and phenotypic data. Tanglegram linking enterococci in phylogenetic tree based on 16S rDNA **(A,B)** and phenotypic traits (I to VII). A different color is used to represent each phenotypic traits and geographical region: The strains labeled in red are strains with probiotic potential. The phylogenetic tree of the 16S sequences were inferred by using maximum likelihood method.

**Figure 4:**
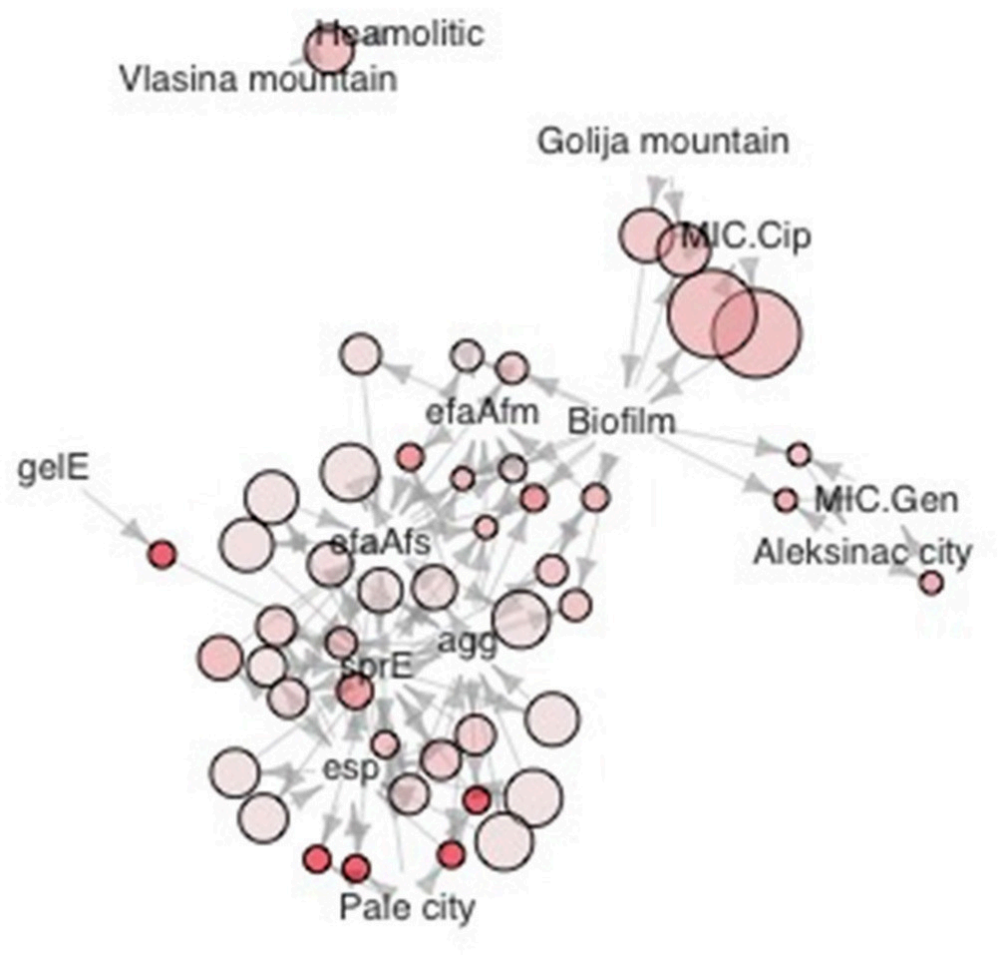
Graphic representation of association rules in the form of network. Size of dots is proportional to the *confidence* measure, color intensity to *adjusted p value*. (Benjamini Hochberg method). Association rules were mined as previously described (Hahsler et al., 2011).

Phylogenetic analysis of 16S rDNA sequences separated selected enterococci isolates into two distinct phylogenetic groups A and B generally following the geographic distribution of the isolates (Figure 3). The broad strain diversity correlated with the diverse origin of the isolates probably due to different environmental pressures, similarly to other LAB strains (Giraffa, 2003; Golic et al., 2012). In addition, the strains were clustered based on phenotypic traits using Jaccard dissimilarity measure resulting in seven distinct groups I-VII (Figure 3), comprising the isolates resistant to gentamicin (I), able to form biofilm and resistant to ciprofloxacin (II), the isolates with virulence genes (III, IV, and V), and the isolates with gelatinase (VI) and hemolytic (VII) activity, respectively. The isolates of phylogenetic group A are correlated with four phenotypic groups. Among them, 54.8% isolates are clustered in group II; 21.4% isolates belong to group VII; 11.9% isolates belong to group VI and 11.9% isolates belong to group I. The isolates belonging to phenotypic groups III, IV, and V, clustered on the basis of the presence of virulence genes, are correlated with the phylogenetic group B. Interestingly, majority of the isolates belonging to the phylogenetic group B (23 out of 33 isolates; 69.7%) are denoted as potential probiotic strains (Figure 3, given in red), based on the results of probiotic tests, presented above. The other members of phylogenetic group B belong to groups I (1 out of 33; 3%), II (4 out of 33; 12.1%) and VII (5 out of 33; 15.2%). The majority of isolates from Aleksinac, belongs to group I (75%). Enterococci from dairy products manufactured at Zlatar Mountain are found mainly in group II (83%), while the isolates from dairy products manufactured in Pale are grouped in group IV (62.5%), II (25%), and V (12.5%). The isolates from Prigorje region belong to groups II (14.3%), VI (43%), and VII (43%). Among all isolates the most persistent phenotypic characteristic is the possibility to produce biofilm with frequency of 0.4. The isolates from Vlasina tend to have more haemolytic properties than it would be expected by pure chance (Figure 4). MIC for gentamicin is associated with dairy products from Aleksinac city, and the same could be said for MIC for ciprofloxacin and Golija Mountain. There is clear co-occurrence of *esp, sprE, agg efaA*^*fs*^*, efaA*^*fm*^ with biofilm formation, with *esp* and *sprE* notably more present in isolates from Pale city.

The correlation between the two trees (phylogenetic and phenotypic) is statistically significant based on Bakers gamma correlation coefficient (0.1039904), which suggests that clustering in phenotypic groups is still under strong influence of phylogenetic relatedness. Depending on the method used p values are *p* = 0.0044 (bootstrap), *p* = 9.4 × 10^−8^ (z-value) and *p* < 0.001 (t-distribution).

In conclusion, to our knowledge, this is the first study combining the data on virulence genes, gelatinase production, haemolytic activity, antibiotic resistance, biofilm production and probiotic features of dairy enterococci isolates, in order to improve our understanding of factors involved in virulence vs. probiotic properties of dairy enterococci. Our results indicate that the virulence genes are sporadically present in tested dairy enterococci isolates and that are not correlated with cytotoxic activity, suggesting that adhesion and biofilm formation are more associated with gut colonization.

## Author contributors

KV, NG–conception and design of the study; NP–performed the main work; KV, AT-V, and JD–participated in the research—virulence traits, antibiotic susceptibility and cytotoxic effect, antimicrobial activity; KV, JD, and MD–participated in the research—gut colonization, adhesion properties and biofilm formation; SM, MT, SB, and NG–participated in the research—relationship between isolates' origins, phylogenetic relatedness and virulence traits; NG, KV, SM, JD, and MT–analyzed, interpreted and critically revised the data; NP, KV, NG, and SM–prepared the manuscript for submission. All authors finally approved the version to be published.

### Conflict of interest statement

The authors declare that the research was conducted in the absence of any commercial or financial relationships that could be construed as a potential conflict of interest.

## Supplementary material

Figure S1 Localities from where the dairy products samples were collected.

Figure S2 Metabolic activity of HT29-MTX cells in the presence of *Enterococcus* sp. strains. The values given in the graph representing the mean values of 3 independent experiments ± standard deviation are expressed in percentages. All experiments were done in triplicates.

Figure S3 Survival of the *Enterococcus* sp. strains in simulated conditions of the gastrointestinal tract. Overnight–time point at the beginning of the experiment when samples were taken from overnight culture; gastric juice–time point when samples were taken from chemically simulated gastric juice; bile salt juice - time point when samples were taken from chemically simulated bile salt juice; intestinum juice–the time point when samples were taken from chemically simulated intestinal juice. Values represent the means of three experiments.

Table S1 The list of strains used in this study (origin, haemolytic and gelatinase activity, biofilm formation).

Table S2 The list of indicator strains used in this study.

Table S3 List of primers used in this study.
