# Supplementary figures and images for: New Insight into Biofilm Formation Ability, the Presence of Virulence Genes and Probiotic Potential of Enterococcus sp. Dairy Isolates
Source: Front Microbiol. 2018 Jan 30;9:78. doi: 10.3389/fmicb.2018.00078 (PMC5797593; doi:10.3389/fmicb.2018.00078)

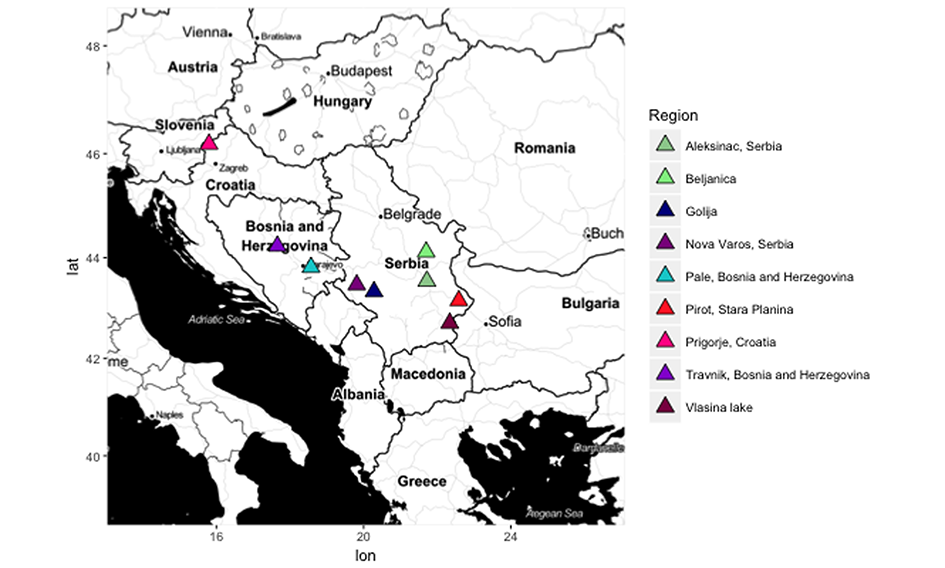

Supplement: Figure S1 — Localities from where the dairy products samples were collected. [file Image1.TIF]

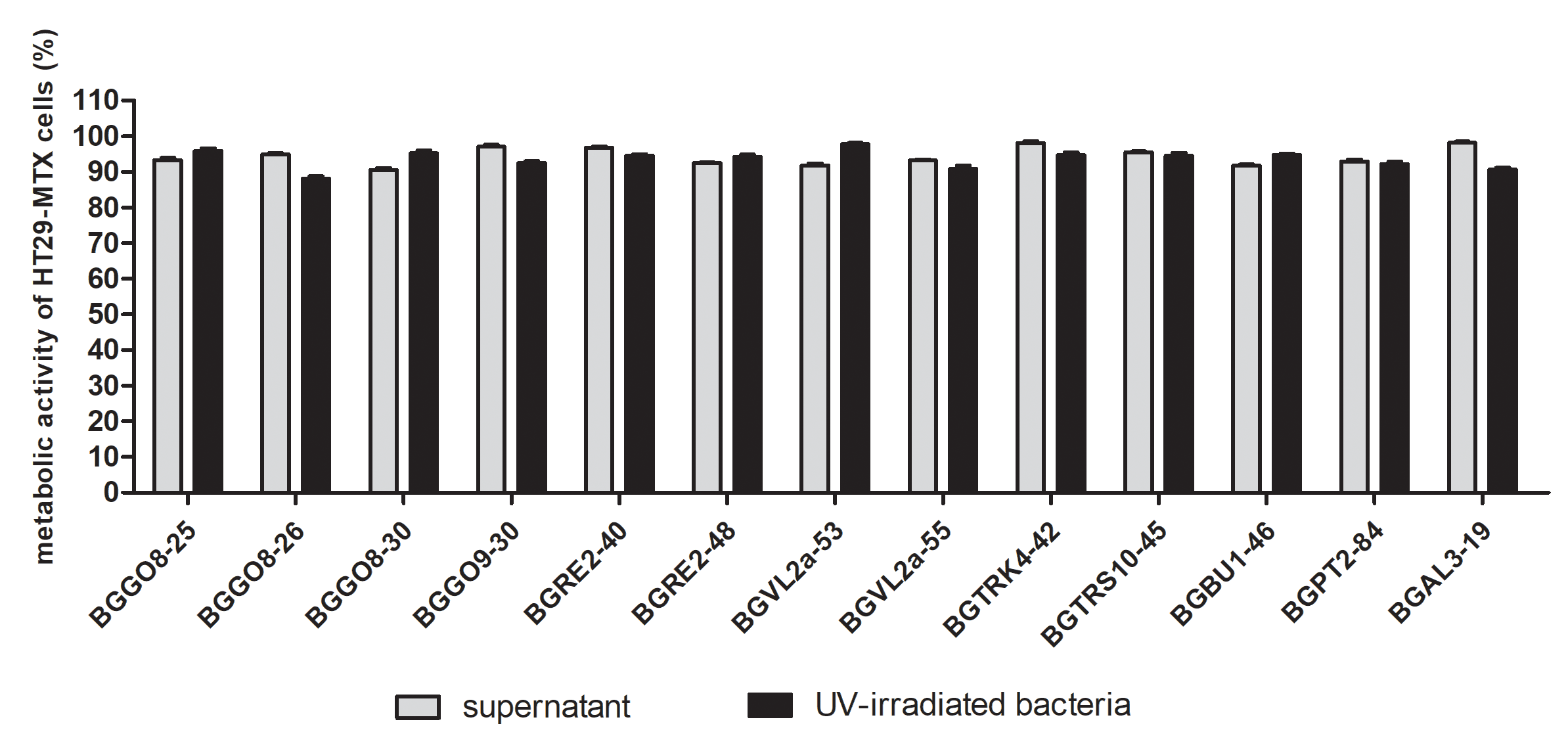

Supplement: Figure S2 — Metabolic activity of HT29-MTX cells in the presence of Enterococcus sp. strains. The values given in the graph representing the mean values of 3 independent experiments ± standard deviation are expressed in percentages. All experiments were done in triplicates. [file Image2.TIF]

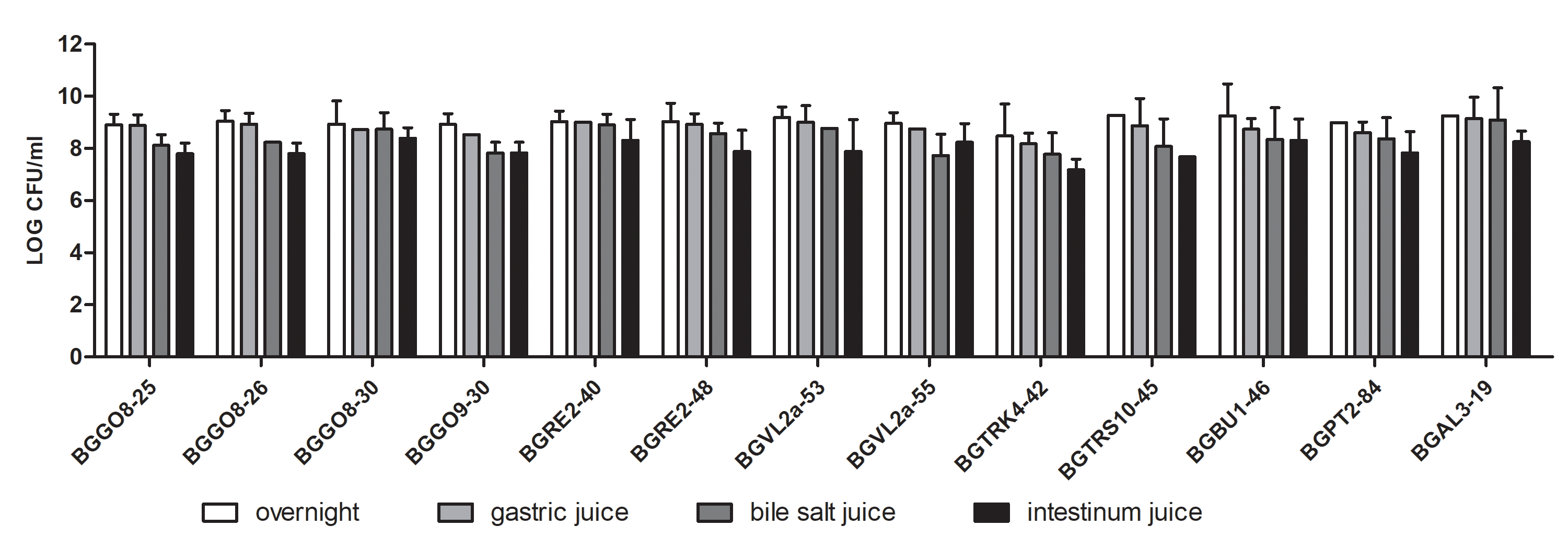

Supplement: Figure S3 — Survival of the Enterococcus sp. strains in simulated conditions of the gastrointestinal tract. Overnight–time point at the beginning of the experiment when samples were taken from overnight culture; gastric juice–time point when samples were taken from chemically simulated gastric juice; bile salt juice - time point when samples were taken from chemically simulated bile salt juice; intestinum juice–the time point when samples were taken from chemically simulated intestinal juice. Values represent the means of three experiments. [file Image3.TIF]
