# Supplementary material for: New Insight into Biofilm Formation Ability, the Presence of Virulence Genes and Probiotic Potential of Enterococcus sp. Dairy Isolates
Source: Front Microbiol. 2018 Jan 30;9:78. doi: 10.3389/fmicb.2018.00078 (PMC5797593; doi:10.3389/fmicb.2018.00078)
Supplement: Table S1 — The list of strains used in this study (origin, haemolytic and gelatinase activity, biofilm formation). [file Table1.DOCX]

**Table S1:** The list of strains used in this study (origin, haemolytic and gelatinase activity, biofilm formation).

| Species/Stain | Region | Origin | | | | Virulence traits | | Biofilm |
| --- | --- | --- | --- | --- | --- | --- | --- | --- |
|  |  | Chesse | Cream | Kajmak | Milk | Heamolitic | Gelatinase |  |
| *En. durans* BGGO1-43 | Serbia, Golija mountain |  |  |  |  |  |  |  |
| *En. durans* BGGO1-44 | Serbia, Golija mountain |  |  |  |  |  |  |  |
| *En. durans* BGGO1-48 | Serbia, Golija mountain |  |  |  |  |  |  |  |
| *En. durans* BGGO1-11 | Serbia, Golija mountain |  |  |  |  |  |  |  |
| *En. durans* BGGO2-47 | Serbia, Golija mountain |  |  |  |  |  |  |  |
| *En. durans* BGGO2-48 | Serbia, Golija mountain |  |  |  |  |  |  |  |
| *En. durans* BGGO5-35 | Serbia, Golija mountain |  |  |  |  |  |  |  |
| *En. durans* BGGO5-13 | Serbia, Golija mountain |  |  |  |  |  |  |  |
| *En. durans* BGGO6-32 | Serbia, Golija mountain |  |  |  |  |  |  |  |
| *En. durans* BGGO6-15 | Serbia, Golija mountain |  |  |  |  |  |  |  |
| *En. faecium* BGGO7-19 | Serbia, Golija mountain |  |  |  |  |  |  |  |
| *En. durans* BGGO8-25 | Serbia, Golija mountain |  |  |  |  |  |  |  |
| *En. durans* BGGO8-26 | Serbia, Golija mountain |  |  |  |  |  |  |  |
| *En. durans* BGGO8-30 | Serbia, Golija mountain |  |  |  |  |  |  |  |
| *En. faecium* BGGO9-28 | Serbia, Golija mountain |  |  |  |  |  |  |  |
| *En. durans* BGGO9-30 | Serbia, Golija mountain |  |  |  |  |  |  |  |
| *En. faecium* BGGO11-27 | Serbia, Golija mountain |  |  |  |  |  |  |  |
| *En. faecium* BGGO11-29 | Serbia, Golija mountain |  |  |  |  |  |  |  |
| *En. durans* BGGO11-38 | Serbia, Golija mountain |  |  |  |  |  |  |  |
| *En. durans* BGGO11-41 | Serbia, Golija mountain |  |  |  |  |  |  |  |
| *En. durans* BGVL2a-50 | Serbia, Vlasina mountain lake |  |  |  |  |  |  |  |
| *En. durans* BGVL2a-53 | Serbia, Vlasina mountain lake |  |  |  |  |  |  |  |
| *En. durans* BGVL2a-54 | Serbia, Vlasina mountain lake |  |  |  |  |  |  |  |
| *En. durans* BGVL2a-55 | Serbia, Vlasina mountain lake |  |  |  |  |  |  |  |
| *En. durans* BGPT2-84 | Serbia, Stara Planina mountain |  |  |  |  |  |  |  |
| *En. durans* BGPT3-30 | Serbia, Stara Planina mountain |  |  |  |  |  |  |  |
| *En. faecium* BGPT5-1P | Serbia, Stara Planina mountain |  |  |  |  |  |  |  |
| *En. durans* BGAL3-13 | Serbia, surrounding Aleksinac city |  |  |  |  |  |  |  |
| *En. durans* BGAL3-16 | Serbia, surrounding Aleksinac city |  |  |  |  |  |  |  |
| *En. durans* BGAL3-19 | Serbia, surrounding Aleksinac city |  |  |  |  |  |  |  |
| *En. durans* BGAL3-23 | Serbia, surrounding Aleksinac city |  |  |  |  |  |  |  |
| *En. durans* BGBU1-40 | Serbia, Beljanica mountain |  |  |  |  |  |  |  |
| *En. durans* BGBU1-46 | Serbia, Beljanica mountain |  |  |  |  |  |  |  |
| *En. durans* BGRE2-40 | Serbia, Beljanica mountain |  |  |  |  |  |  |  |
| *En. hirae* BGRE2-48 | Serbia, Beljanica mountain |  |  |  |  |  |  |  |
| *En. faecium* BGZLM1-5 | Serbia, Zlatar mountain |  |  |  |  |  |  |  |
| *En. durans* BGZLS30-21 | Serbia, Zlatar mountain |  |  |  |  |  |  |  |
| *En. faecium* BGZLS30-22 | Serbia, Zlatar mountain |  |  |  |  |  |  |  |
| *En. faecalis* BGZLS45-5 | Serbia, Zlatar mountain |  |  |  |  |  |  |  |
| *En. faecalis* BGZLS45-13 | Serbia, Zlatar mountain |  |  |  |  |  |  |  |
| *En. faecalis* BGZLS45-20 | Serbia, Zlatar mountain |  |  |  |  |  |  |  |
| *En. durans* BGTRS1-10 | Bosnia and Herzegovina, Vlašić mountain |  |  |  |  |  |  |  |
| *En. durans* BGTRS1-20 | Bosnia and Herzegovina, Vlašić mountain |  |  |  |  |  |  |  |
| *En. durans* BGTRS1-29 | Bosnia and Herzegovina, Vlašić mountain |  |  |  |  |  |  |  |
| *En. durans* BGTRS1-49 | Bosnia and Herzegovina, Vlašić mountain |  |  |  |  |  |  |  |
| *En. durans* BGTRS7-67 | Bosnia and Herzegovina, Vlašić mountain |  |  |  |  |  |  |  |
| *En. durans* BGTRS7-54 | Bosnia and Herzegovina, Vlašić mountain |  |  |  |  |  |  |  |
| *En. durans* BGTRS10-45 | Bosnia and Herzegovina, Vlašić mountain |  |  |  |  |  |  |  |
| *En. durans* BGTRM1-42 | Bosnia and Herzegovina, Vlašić mountain |  |  |  |  |  |  |  |
| *En. durans* BGTRM1-52 | Bosnia and Herzegovina, Vlašić mountain |  |  |  |  |  |  |  |
| *En. durans* BGTRM7-33 | Bosnia and Herzegovina, Vlašić mountain |  |  |  |  |  |  |  |
| *En. durans* BGTRM7-39 | Bosnia and Herzegovina, Vlašić mountain |  |  |  |  |  |  |  |
| *En. durans* BGTRM7-40 | Bosnia and Herzegovina, Vlašić mountain |  |  |  |  |  |  |  |
| *En. durans* BGTRM7-43 | Bosnia and Herzegovina, Vlašić mountain |  |  |  |  |  |  |  |
| *En. durans* BGTRM7-47 | Bosnia and Herzegovina, Vlašić mountain |  |  |  |  |  |  |  |
| *En. faecalis* BGTRM10-21 | Bosnia and Herzegovina, Vlašić mountain |  |  |  |  |  |  |  |
| *En.italicus* BGTRK1-35 | Bosnia and Herzegovina, Vlašić mountain |  |  |  |  |  |  |  |
| *En. italicus* BGTRK4-35 | Bosnia and Herzegovina, Vlašić mountain |  |  |  |  |  |  |  |
| *En. italicus* BGTRK4-42 | Bosnia and Herzegovina, Vlašić mountain |  |  |  |  |  |  |  |
| *En. durans* BGTRK10-29 | Bosnia and Herzegovina, Vlašić mountain |  |  |  |  |  |  |  |
| *En. faecium* BGPAS1-2 | Bosnia and Herzegovina, Pale mountain city |  |  |  |  |  |  |  |
| *En. faecium* BGPAS1-3 | Bosnia and Herzegovina, Pale mountain city |  |  |  |  |  |  |  |
| *En. faecium* BGPAS1-4 | Bosnia and Herzegovina, Pale mountain city |  |  |  |  |  |  |  |
| *En. faecium* BGPAS1-10 | Bosnia and Herzegovina, Pale mountain city |  |  |  |  |  |  |  |
| *En. faecium* BGPAS1-20 | Bosnia and Herzegovina, Pale mountain city |  |  |  |  |  |  |  |
| *En. faecium* BGPAS1-48 | Bosnia and Herzegovina, Pale mountain city |  |  |  |  |  |  |  |
| *En. faecium* BGPAS1-58 | Bosnia and Herzegovina, Pale mountain city |  |  |  |  |  |  |  |
| *En. faecium* BGPAS1-71 | Bosnia and Herzegovina, Pale mountain city |  |  |  |  |  |  |  |
| *En. durans* BGZG1-18 | Croatia, Prigorje region |  |  |  |  |  |  |  |
| *En. durans* BGZG2-1 | Croatia, Prigorje region |  |  |  |  |  |  |  |
| *En. durans* BGZG2-8 | Croatia, Prigorje region |  |  |  |  |  |  |  |
| *En. faecalis* BGZG2-9 | Croatia, Prigorje region |  |  |  |  |  |  |  |
| *En. faecalis* BGZG2-13 | Croatia, Prigorje region |  |  |  |  |  |  |  |
| *En. durans* BGZG2-20 | Croatia, Prigorje region |  |  |  |  |  |  |  |
| *En. durans* BGZG4-19 | Croatia, Prigorje region |  |  |  |  |  |  |  |
