# Supplementary material for: New Insight into Biofilm Formation Ability, the Presence of Virulence Genes and Probiotic Potential of Enterococcus sp. Dairy Isolates
Source: Front Microbiol. 2018 Jan 30;9:78. doi: 10.3389/fmicb.2018.00078 (PMC5797593; doi:10.3389/fmicb.2018.00078)
Supplement: Table S2 — The list of indicator strains used in this study. [file Table2.DOCX]

**Table S2:** The list of indicator strains used in this study

| Bacterial strains | Source |
| --- | --- |
| *Lactococcus lactis* subsp. *lactis* BGMN1-596 | Laboratory collection |
| *Enterococcus faecalis* BG221 | Laboratory collection |
| *Enterococcs faecalis* V583 | Kindly provided by Pousen |
| *Listeria monocytogenes* ATCC 19111 | ATCC ^a^ |
| *Staphylococcus aureus* ATCC 25923 | ATCC |
| *Escherichia coli* ATCC 25922 | ATCC |
| Salmonella Enteritidis 654/7E | Veterinary isolate kindly provided by Scientific Veterinary Institute ‘Novi Sad’, Serbia |

^a^ ATCC-American Type Culture Collection, Manassas, VA, USA.
