# Supplementary material for: New Insight into Biofilm Formation Ability, the Presence of Virulence Genes and Probiotic Potential of Enterococcus sp. Dairy Isolates
Source: Front Microbiol. 2018 Jan 30;9:78. doi: 10.3389/fmicb.2018.00078 (PMC5797593; doi:10.3389/fmicb.2018.00078)
Supplement: Table S3 — List of primers used in this study. [file Table3.DOCX]

**Table S3:** List of primers used in this study.

| Genes | Primers | Product size | T°C | Reference |
| --- | --- | --- | --- | --- |
| Virulence factors | | | | |
| *gelE* | 5′-CGGAAGGCGTTACTGTTGAT-3′  5′-GAGCCATGGTTTCTGGTTGT-3′ | 957 bp | 46°C | This study |
| *sprE* | 5′- TTGAGCTCCGTTCCTGCCGAAAGTCATTC-3′  5′-TTGGTACCGATTGGGGAACCAGATTGACC-3′ | 591 bp | 58°C | (Nakayama *et al.*, 2002) |
| *ace* | 5′-AAAGTAGAATTAGATCCACAC-3′  5′-TCTATCACATTCGGTTGCG-3′ | 320 bp | 56°C | (Duprè *et al.*, 2003) |
| *hylN* | 5′-ACAGAAGAGCTGCAGGAAATG-3′  5′-GACTGACGTCCAAGTTTCCAA-3′ | 276 bp | 56°C | (Vankerckhoven *et al.*, 2004) |
| *agg* | 5′-AAGAAAAAGAAGTAGACCAAC-3′  5′-AAACGGCAAGACAAGTAAATA-3′ | 1553 bp | 54°C | (Eaton and Gasson, 2001) |
| *cylA* | 5′-TGGATGATAGTGATAGGAAGT-3′  5′-TCTACAGTAAATCTTTCGTCA-3′ | 517 bp | 58°C | (Eaton and Gasson, 2001) |
| *esp* | 5′-TTGCTAATGCTAGTCCCAGACC-3′  5′-GCGTCAACACTTGCATTGCCGAA-3′ | 933 bp | 58°C | (Eaton and Gasson, 2001) |
| *efaA^fs^* | 5′-GACAGACCCTCACGAATA-3′  5′-ATGTCATCATGCTGTAGTA-3′ | 705 bp | 56°C | (Eaton and Gasson, 2001) |
| *efaA^fm^* | 5′-AACAGATCCGCATGAATA-3′  5′-CATTTCATCATCTGATAGTA-3′ | 735 bp | 56°C | (Eaton and Gasson, 2001) |
| *fsrA* | 5′-ATGAGTGAACAAATGGCTATTTA-3′  5′-CTAAGTAAGAAATAGTGCCTTGA-3 | 740 bp | 43°C | (Nakayama *et al.*, 2002) |
| *fsrB* | 5′-GGGAGCTCTGGACAAAGTATTATCTAACCG-3′  5′-TTGGTACCCACACCATCACTGACTTTTGC-3′ | 566 bp | 43°C | (Nakayama *et al.*, 2002) |
| *fsrC* | 5′-ATGATTTTGTCGTTATTAGCTACT-3′  5′-CATCGTTAACAACTTTTTTACTG-3′ | 1343 bp | 43°C | (Nakayama *et al.*, 2002) |
| Bacteriocins | | | | |
| *entA* | 5'-AAATATTATGGAAATGGAGTGTAT-3'  5'-GCACTTCCCTGGAATTGCTC-3' | 126 bp | 58°C | (Toit *et al.*, 2000) |
| *entB* | 5'-GAAAATGATCACAGAATGCCTA-3'  5'-GTTGCATTTAGAGTATACATTTG-3' | 162 bp | 56°C | (Toit *et al.*, 2000) |
| *entP* | 5'-TATGGTAATGGTGTTTATTGTAAT-3'  5'-ATGTCCCATACCTGCCAAAC-3' | 120 bp | 56°C | (Toit *et al.*, 2000). |
| *entL* | 5'-GGACAACAATTCGCGGAAACACT-3'  5'-GCCAAGTAAAGGTAGAATAAA-3' | 1007 bp | 55°C | (Nilsen *et al.*, 2003) |
| *entL50* | 5'-STGGGAGCAATCGCAAAATTAG-3'  5'-ATTGCCCATCCTTCTCCAAT-3' | 98 bp | 56°C | (Toit *et al.*, 2000) |
| *entAS48* | 5'-GAGGAGTITCATGATTAAAGA-3'  5'-CATATTGTTAAATTACCAAGCAA-3' | 340 bp | 54°C | (Toit *et al.*, 2000) |
| *ent1070* | 5'-CCTATTGGGGGAGAGTCGGT-3'  5'-ATACATTCTTCCACTTATTTTT-3' | 343 bp | 56°C | (Omar *et al.*, 2004) |
| *bac31* | 5'-TATTACGGAAATGGTTTATATTGT-3'  5'-TCTAGGAGCCCAAGGGCC-3' | 123 bp | 58°C | (Toit *et al.*, 2000) |
